# Changes in the prevalence of the common risk factors for non-communicable diseases in Uganda between 2014 and 2023: Informed by nationally representative cross-sectional surveys

**DOI:** 10.1371/journal.pgph.0003755

**Published:** 2025-04-08

**Authors:** Ronald Kusolo, Gerald N. Mutungi, Mary Mbuliro, Richard Kajjura, Ronald Wesonga, Silver K. Bahendeka, David Guwatudde

**Affiliations:** 1 Department of Epidemiology and Biostatistics, School of Public Health, Makerere University, Kampala, Uganda; 2 Department of Non-Communicable Diseases, Ministry of Health, Kampala, Uganda; 3 Department of Community Health and Behavioral Sciences, School of Public Health, Makerere University, Kampala, Uganda; 4 Department of Statistics, College of Science, Sultan Qaboos University, Muscat, Sultanate of Oman; 5 Department of Internal Medicine, School of Medicine Uganda Martyrs’ University, Nkozi, Uganda; 6 Department of Diabetes & Endocrinology, St. Francis Hospital, Kampala, Uganda; Indian Council of Medical Research, INDIA

## Abstract

Non-communicable diseases (NCDs) remain the biggest contributor to global mortality. An important way to control NCDs is to focus on reducing the prevalence of the common NCD risk factors for better NCD prevention planning. Uganda conducted its first nationally representative NCD risk factor survey in 2014, and a second in 2023. We analyzed the prevalence of the common NCD risk factors to assess changes in these between 2014 and 2023. Both surveys drew countrywide samples, and the World Health Organization’s STEPS tool was used to collect the data. We calculated weighted prevalence of the following NCD risk factors: high blood pressure, high blood glucose, overweight and obesity, current alcohol consumption, current tobacco use, inadequate consumption of fruits and vegetables, inadequate physical activity, and sedentariness. The 2014 survey enrolled 3987 participants, whereas the 2023 survey enrolled 3694. The risk factor prevalences that increased significantly were: high blood glucose from 1.5% in 2014 to 3.3% in 2023 (p< 0.001); overweight and obesity from 19.3% in 2014 to 24.1% in 2023 (p< 0.001); current alcohol consumption from 28.5% in 2014 to 31.1% in 2023 (p=0.013); and sedentariness from 26.6% in 2014 to 31.9% in 2023 (p< 0.001). The risk factor prevalences that decreased significantly were: inadequate physical activity from 5.0% in 2014 to 3.6% in 2023 (p=0.003), and current smoke tobacco use from 9.6% in 2014, to 8.3% in 2023 (p= 0.046). No significant changes were observed in the prevalence of high blood pressure from 24.6% in 2014 to 25.4% in 2023 (p= 0.418), and inadequate consumption of fruits and vegetables from 87.8% in 2014 to 86.4% in 2023 (p=0.067). There is an urgent need for various stakeholders in Uganda to implement interventions targeting reduction in the prevalence of NCD risk factors to prevent the increasing burden of NCDs and associated mortality.

## Introduction

Non-communicable diseases (NCDs), mainly cardiovascular diseases (heart disease and stroke), cancers, diabetes, chronic respiratory diseases, and mental health disorders, kill approximately 41 million people each year, equivalent to 74% of all deaths globally. Of these, 17 million are premature deaths that occur before the age of 70 years; and 86% occur in low- and middle-income countries (LMIC) [1]. Thus, NCDs are the greatest contributor to global mortality. Currently within the African region, cardiovascular diseases, cancers, diabetes mellitus and chronic respiratory diseases account for over 70% of NCD-related mortality [2].

An important way to control NCDs is to focus on reducing the prevalence of risk factors associated with these diseases [1]. Surveillance, or regular monitoring of the common risk factors for NCDs is essential in public health to inform decision making for prevention and control of NCDs. Indeed the WHO recommends that national surveys to update the prevalence of the common NCD risk factors should be conducted approximately every 5 years [3]. The common NCD risk factors can broadly be categorized into two, i.e., the behavioral risk factors comprising physical inactivity, alcohol consumption, tobacco use, low consumption of fruits and vegetables, and sedentariness; and physiological/ metabolic risk factors comprising overweight and obesity, high blood pressure, hyperglycemia (high blood glucose levels), and hyperlipidemia (high levels of fat in the blood) [1]. A number of LMICs have now conducted nationally representative population based surveys that collect data on the common risk factors for NCDs, mostly using the WHO STEPwise approach to NCD risk factor surveillance (STEPS) methodology [4]. Findings from these surveys can be used not only to monitor changes in the prevalence of the common NCD risk factors, but to also inform NCD prevention planning, and/or evaluate the impact of any prevention interventions that may have been implemented.

Uganda conducted its first nationally representative NCD risk factor survey in 2014 [5–7], and a second in 2023 [8]. Since 2014, Uganda’s Ministry of Health has spearheaded a number of public health preventive initiatives aimed at reducing the general public’s risk for NCDs, including amongst others, the presidential initiative on healthy eating and healthy lifestyles [9], development of the NCD Multi-Sectoral Strategic Plan [10], and launching a National Day of Physical activity in 2018 - held annually to raise awareness about the growing problem of NCDs in Uganda [11]. It is however not known if these have made any impact yet, on the prevalence of the common NCD risk factors. Of public health interest is to monitor changes in the risk factors for better NCD prevention planning. We therefore analyzed the prevalence of the common NCD risk factors using data from the Uganda national NCD risk factor surveys conducted in 2014, and that conducted in 2023. The primary objective of our analysis was to assess any changes in the prevalence of the common NCD risk factors between 2014 and 2023.

## Materials and methods

We used the same methodology to conduct the 2014, and the 2023 surveys, using the WHO STEPS survey guidelines [12]. Below we describe in details the specific methods used.

### Study design

A cross-sectional study design was used to conduct both the 2014 and the 2023 surveys. The 2014 survey was conducted between March and July 2014, whereas the 2023 survey was conducted between February and April 2023.

### Sample size

Sample size for both the 2014 and the 2023 surveys were calculated assuming a 50% prevalence of the risk factors (*P* = 0.5, which is the recommended value for unknown prevalence, and gives the most conservative sample size), a 5% level of significance (α =0.05), a margin of error of 0.05 (δ =0.05); an expected response rate of 80%, six age-sex categories (18-29, 30-45, 45-69, each by male and female), and a 1.5 design effect. Using these parameters, the sample size calculation was made using the WHO STEPS sample size calculator [13]. The calculated sample size for the 2014 survey was 4900, and that for the 2023 survey was 4340, each in respect to the available Enumeration Areas (EAs). During the 2023 survey, a large geographical area had been taken over by the Uganda Military after 2014 and was not included in the survey, thus the smaller sample size.

### Study population & sampling

Uganda currently has a total population of approximately 45.8 million [14], of which approximately 45% are adults aged 18 years or older. Both surveys covered the whole country, and a three stage stratified sampling was used as described below. We used the population enumeration areas (EAs) information that was provided by the Uganda Bureau of Statistics (UBOS). UBOS had demarcated the whole country into a total of 78,691 EAs that they used for the Uganda Population and Housing Census of 2014. The strata were regions stratified by urban-rural locations, that is, regions (Northern, Western, Central & Eastern), then by urban and rural location within each region, generating a total of 8 strata. The first stage of selecting the study sample was a random sample of 350 enumeration areas (EA) in 2014, and 310 in 2023 from the 8 strata using proportion to size sampling (PPS), as the first stage of sampling. Research Assistants (RA) that had received a five-day training on household listing procedures were then dispatched throughout the country to list all households within the sampled EAs. The listing was used to conduct the second stage of sample selection by randomly selecting 14 households within each EA.

A different team (Interviewers), who had also received a five-day training on WHO STEPwise approach to NCD risk factor surveillance, STEPS data collection procedures and ethics among others was then dispatched throughout the country. They started by enumerating all eligible members in the sampled households and recorded these in an electronic tablet. The final third stage of sample selection involved randomly selecting one eligible household member within each household, which was conducted by the study Interviewers using an android tablet that was pre-set to randomly select one individual from the list of household members for inclusion in the survey, leading to a total sample of 4900 in 2014, and 4340 in 2023. Sample selection was conducted without replacement, thus there was no replacement of the android tablet pre-selected household member to prevent potential selection bias.

Eligible participants were adults aged between 18-69 years of age, had been members of their household for at least six months preceding the date of the survey, of sound mind, and able to give written informed consent. Household members with the following characteristics were excluded: unable to stand without support (e.g., after limb amputation without a prosthesis), clearly under the influence of alcohol or other drug(s) abuse, and moribund and obtundent.

### Measurements

Both surveys used the World Health Organization’s (WHO) STEPwise approach to surveillance, a standardized method of analyzing risk factors for NCDs [4]. The STEPwise approach is a sequential process starting with collecting data on key risk factors using a questionnaire (STEP 1), followed by physical measurements (STEP 2), and finally collection of biological samples for biochemical assessments (STEP 3).

Participants were informed that they would participate in the survey for 2 days: Day 1 for STEP 1 and STEP 2, and day 2 for STEP 3. In STEP 1, data were collected on demographic and behavioral information using the STEPS questionnaire [12]. This step collected information on the demographic and social characteristics (e.g., age, sex, level of education, employment, income, etc.); behavioral characteristics (e.g., tobacco use, alcohol consumption, fruit and vegetable consumption, physical activity, etc.). It also included information on health history like history of raised blood pressure, raised blood glucose, raised blood cholesterol, cardiovascular diseases, lifestyle advice and cervical cancer screening for women respondents, household energy use, mental health (depression) and impact of the COVID-19 pandemic on behavioral risk factors.

STEP 2 involved making the physical measurements including height, weight, girth (waist, hip), blood pressure and pulse, which were made immediately after administering the questionnaire. Body weight was measured to the nearest 0.1 kg using a calibrated digital weighing scale (SECA® 877), while the subject was barefoot and wearing light clothing. Standing height was measured using a portable stadiometer (SECA® 877) to the nearest 0.5 cm with the subject standing barefoot, back square against the stadiometer and eyes looking straight ahead. Waist and hip circumferences (to the nearest 0.5 cm) were measured using a non-stretchable standard tape measure and measurement taken midway between the lowest rib and the iliac crest with the subject standing at the end of gentle expiration and hip measurement at the level of the greater trochanters. Three blood pressure readings were taken 3–5 minutes apart. Blood pressure measurements were taken on the left arm with the participant in the sitting position using battery powered digital blood pressure machine (Boso Medicus Uno1). Heart rate reading was also recorded. Steps 1 and 2 measurements were conducted in the participant’s home.

After administering the questionnaire and making the physical measurements, Interviewers requested participants to converge at pre-arranged location the following morning to conduct STEP 3 measurements. Participants were requested to fast from food and drinks for at least eight hours overnight, and not to indulge in exercise or smoking in the morning prior to the measurements. Plain water could be consumed. Participants converged at the agreed location in the EA starting from 7.00 am. Most of the participants had a very short distance to walk, and if substantial distance was envisaged, they came on a motorcycle (Boda-Boda) or were collected by the survey team vehicle.

Thus STEP 3 measurements were conducted in a fasting state, and involved performing biochemical measurements using point of care devices supplied to the study team by the WHO, and accuracy of which had already been validated. A blood sample from a finger prick was taken for the measurement of total cholesterol (TC), high-density lipoprotein cholesterol (HDL-C), and fasting blood glucose (FBG). Analysis carried out using the CardioChek PA meter (Miller Medical Supplies). The finger was first cleaned with cotton using plain water and then dried with dry cotton. A single-use safety lancet (Unistik 3, Comfort; Owen Mumford) was then used to prick the finger. Only participants that reported compliance with the procedures for Step 3 (overnight 8-h fast, no exercise and no smoking in the morning of the study) were eligible for finger-prick blood sample collection. Only in the 2023 survey, a spot urine sodium, creatinine and cotinine testing were measured.

### Ethics

Both the 2014 and 2023 the surveys were approved by the Research and Ethics Committee (REC) of Nsambya St. Francis Hospital, Kampala, Uganda (Approval #: IRC/PRJ/11/13/031, Approval #: SFHN-2022-43, respectively). Written informed consent was obtained from eligible subjects before enrollment in the study. Participants with at least two systolic blood pressure readings of at least 121 mm Hg, and/or diastolic blood pressure of at least 81mm Hg, and/or with fasting plasma glucose of at least 6.1 mmol/L, and were not already on treatment for hypertension and/or diabetes, were advised to as soon as possible report to the nearest government owned health facility for further evaluation.

### Data management & analysis

#### Data management.

The STEPS data collection tool was programmed in the Open Data Kit (ODK) software, an open-source data collection platform. All data was collected through a password protected tablet to ensure confidentiality and encrypted during transmission from the Interviewers to the server to protect data from unauthorized access. Data quality indicators were monitored through a real-time dashboard by the study Data Manager. All the identified quality issues were discussed with the field team for timely resolution. The final cleaned datasets were then merged and locked for analysis.

#### Statistical analysis methods.

The response rate was calculated as the proportion of eligible household members who consented to participate in the survey, out of the total number of sampled households. Descriptive statistics are reported as means with corresponding standard deviations for continuous variables like age, and categorical variables, including sex, urban-rural status and region of residence, level of education, marital status, and employment status are reported as frequencies and proportion/percentages.

The prevalence of the common NCD risk factors are reported by region, and by urban-rural location (urbancity), comparing their prevalence between the 2014 and the 2023 surveys. Below is a detailed description of how the prevalence of the risk factors were calculated.

#### Behavioral risk factors.

i) The prevalence of current alcohol consumption was calculated as the percentage of participants reporting to had consumed any amount and type of alcohol in the past 30 days preceding the day of the interview.
ii) The prevalence of current tobacco use was calculated using two different indicators. The first was prevalence of current use of all forms of tobacco, calculated as the percentage of participants reporting to had used smoke and/or smokeless tobacco in the past 30 days, including smoking, chewing, and/or sniffing. The second indicator was prevalence of current use of smoke tobacco, calculated as the percentage of participants reporting to had smoked tobacco in the past 30 days.
iii) The prevalence of inadequate consumption of fruits and vegetables was calculated as the percentage of participants reporting to had consumed an average of less than five servings of fruits and/or vegetables combined per day, over the past seven days preceding the day of interview.
iv) Prevalence of inadequate physical activity was calculated as the percentage of participants reporting less than 150 minutes per week of moderate intensity physical activity, and/or less than 75 minutes per week of vigorous intensity physical activity, per recommendations by the WHO [15].
v) The prevalence of sedentariness was calculated as the percentage of participants reporting an average of more than four hours of awake time, seated, reclined or lying posture. The cut-off of four hours was based on a study that showed that more than four hours of sedentary increased the risk of all-cause cardiovascular and cancer mortality, and incident type 2 diabetes [16].

#### Physiological/ Metabolic risk factors.

i) The prevalence of high blood pressure was calculated as the percentage of participants with an average of two readings of systolic blood pressure ≥ 140, and/or an average of two readings of diastolic blood pressure ≥ 90 mmHg [17,18], and/or reporting to currently be on hypertension treatment prescribed by a health care worker.
ii) The prevalence of high blood glucose was calculated as the percentage of participants with a fasting plasma glucose reading of > 7.0 millimoles per liter (mmol/L), or reporting to currently be on anti-diabetes treatment prescribed by a health care worker [19].
iii) The prevalence of overweight and obesity was calculated as a Body Mass Index (BMI) greater than, or equal to 25. BMI for a participant was calculated as weight in kilograms per squared meter (kg/m^2^) [20].

We report weighted prevalence estimates using the study sampling weights. Three distinct sampling weights were applied in calculating the prevalence of the above risk factors. Step 1 weights were used for the self-reported behavioral risk factors including: prevalence of current alcohol consumption, prevalence of current tobacco use (except cotinine/ nicotine test), prevalence of inadequate consumption of fruits and vegetables, prevalence of sedentariness, and prevalence of inadequate physical activity. Step 2 weights were applied in calculating the prevalence of physiological/ metabolic NCD risk factors, including prevalence of high blood pressure, prevalence of high blood glucose, and the prevalence of overweight and obesity.

Chi-square tests were applied to assess statistical differences in the prevalence of the NCD risk factors between the 2014, and the 2023 surveys, with a significance level at 5%. Data analysis was conducted using the STATA software version 15.

## Results

### Response rates in the 2023 survey

For the 2014 survey, out of the 4900 nationwide sampled subjects, 3987 consented to participate in the survey, giving a response rate of 81.4%. For the 2023 survey, out of the 4340 nationwide sampled subjects, 3694 consented to participate in the survey, giving a response rate of 85.1%. We observed no statistically significant differences in the response rates by sex or by region between the two surveys.

### Characteristics of the participants

Of the 3987 participants in the 2014 survey, 2383 (59,8%) were female, 2903 (72.8%) were rural residents, 1343 (33.7%) were aged at least 40 years, and 3333 (83.6%) had completed at least primary school education. By region of residence, in the 2014 survey 779 (19.5%) were sampled from the northern region compared to 810 (21.9%) in 2023; 1294 (32.4%) were sampled from the central region in 2014 compared to 904 (24.5%) in 2023; 964 (24.2%) were sampled from the eastern in 2014 compared to 1052 (28,5%) in 2023; and 950 (23.8%) were sampled from the western region compared to 928 (25.1%) in 2023. The average age of participants in the 2014 survey was 35.4 (SD=13.0) compared to 38.3 (SD=13.8) in the 2023 survey. A summary of these, and other selected characteristics of the participants is presented in Table 1.

**Table 1:** Participant characteristics.

Characteristic		Survey Year			
		2014		2023	
		-n-	(Un weighted %)	-n-	(Un weighted %)
All participants	–	3987	100.0	3,694	100.0
Sex:	Males	1,604	40.2	1,426	38.6
	Females	2,383	59.8	2,268	61.4
Urban-rural:	Rural	2,903	72.8	2,892	78.3
	Urban	1,084	27.2	802	21.7
Region:	Northern	779	19.5	810	21.9
	Central	1,294	32.5	904	24.5
	Eastern	964	24.2	1,052	28.5
	Western	950	23.8	928	25.1
Age in years:	18-19	307	7.7	186	5.0
	20-29	1,311	32.9	1,033	28.0
	30-39	1,026	25.7	915	24.8
	≥40	1,343	33.7	1,560	42.2
	Mean ± SD	3,987	35.4 ±13.0	3,694	38.3 ±13.8
Level of education:	None	654	16.4	2,122	57.4
	Primary school	1,626	40.8	968	26.2
	Secondary school	1,317	33.0	463	12.5
	University or higher	374	9.4	140	3.9
	Not stated	16	0.4	1	0
Marital status:	Never married	627	15.7	455	12.3
	Currently married	2,550	64.0	2,033	55.0
	Separated	317	8.0	414	11.2
	Divorced	89	2.2	38	1.0
	Widowed	306	7.7	268	7.3
	Cohabiting	94	2.4	485	13.1
	Refused	3	0.1	1	0
Employment status:	Employed	2,601	65.3	2941	79.6
	Non-paid	45	1.1	177	4.8
	Student	182	4.6	64	1.7
	Unemployed	1,157	29.0	511	13.8
	Refused	1	0	1	0

### Prevalence of the common NCD risk factors

#### Current alcohol consumption.

The prevalence of current alcohol consumption increased significantly from 28.5% in 2014, to 31.1% in 2023 (p=0.013). The significant increases occurred in all regions of the country except the Central region (p= 0.149) – Table 2. Significant increases in the prevalence of current alcohol consumption were also observed in both rural and urban areas between the 2014 survey and the 2023 survey (Table 3).

**Table 2:** Prevalence of the common NCD risk factors by region.

Risk Factor	Region												Overall		
	Northern			Central			Eastern			Western					
	2014	2023	p-value	2014	2023	p-value	2014	2023	p-value	2014	2023	p-value	2014	2023	p-value
Current alcohol consumption	36.9%	41.1%	0.086	28.9%	26.1%	0.149	20.8%	26.4%	0.003	28.2%	34.8%	0.002	28.5%	31.1%	0.013
Current use of all forms of tobacco	19.6%	19.5%	0.959	7.0%	6.7%	0.785	7.5%	8.3%	0.506	12.0%	9.1%	0.041	11.2%	10.1%	0.119
Current smoke tobacco use	14.1%	16.3%	0.222	6.5%	5.5%	0.335	6.8%	6.3%	0.650	11.6%	8.1%	0.011	9.6%	8.3%	0.046
Inadequate consumption of fruits and vegs/ day	87.7%	85.2%	0.146	91.7%	83.8%	<0.001	75.1%	82.5%	<0.001	95.0%	94.4%	0.562	87.8%	86.4%	0.067
Inadequate physical activity	3.9%	1.5%	0.003	7.5%	4.4%	0.003	5.6%	4.4%	0.216	2.9%	3.2%	0.705	5.0%	3.6%	0.003
Sedentariness	27.5%	30.9%	0.136	33.5%	38.9%	0.009	29.8%	32.3%	0.226	16.3%	25.1%	<0.001	26.6%	31.9%	<0.001
High blood pressure	19.4%	21.0%	0.427	28.1%	28.7%	0.759	26.3%	28.0%	0.392	23.6%	21.9%	0.379	24.6%	25.4%	0.418
High blood glucose	1.3%	2.2%	0.172	2.1%	5.2%	<0.001	1.1%	3.0%	0.003	1.5%	2.7%	0.030	1.5%	3.3%	<0.001
Overweight and obesity	8.3%	14.6%	<0.001	30.2%	36.8%	0.001	16.8%	19.3%	0.145	19.3%	24.0%	0.013	19.3%	24.1%	<0.001

**Table 3:** Prevalence of the common NCD risk factors by Urbanicity.

Risk Factors	Type of Location						Overall		
	Urban			Rural					
	2014	2023	p-value	2014	2023	p-value	2014	2023	p-value
Current alcohol consumption	22.8%	28.2%	0.008	29.9%	32.4%	0.039	28.5%	31.1%	0.013
Current use of all forms of tobacco	6.7%	7.8%	0.359	12.2%	11.1%	0.192	11.2%	10.1%	0.119
Current use of smoke tobacco	6.2%	7.1%	0.436	10.4%	8.9%	0.053	9.6%	8.3%	0.046
Inadequate consumption of fruits and vegs/ day	89.4%	86.5%	0.054	87.4%	86.4%	0.259	87.8%	86.4%	0.067
Inadequate physical activity	8.4%	6.5%	0.124	4.2%	2.3%	<0.001	5.0%	3.6%	0.003
Sedentariness	34.6%	34.4%	0.928	24.7%	30.7%	<0.001	26.6%	31.9%	<0.001
High blood pressure	26.3%	29.9%	0.085	24.2%	23.4%	0.475	24.6%	25.4%	0.418
High blood glucose	2.3%	3.6%	0.094	1.3%	3.2%	<0.001	1.5%	3.3%	<0.001
Overweight and obesity	32.3%	33.7%	0.522	16.2%	19.9%	<0.001	19.3%	24.1%	<0.001

#### Current use of all forms of tobacco.

Overall, no significant differences were observed in current use of all forms of tobacco between 2014 and 2023, only a marginal significant decrease was observed in the Western region from 12.0% in 2014, to 9.1% in 2023 (p= 0.041). No significant differences were observed in the prevalence of current use of all forms of tobacco in urban, or in rural areas (Table 3).

#### Current use of smoke tobacco.

There was an overall significant decrease in the prevalence of current use of smoke tobacco from 9.6% in 2014, to 8.3% in 2023 (p= 0.046). This significant decrease occurred in the Western region of the country only from 11.6% in 2014, to 8.1% in 2023 (p= 0.011), Table 2. No significant differences were observed in the prevalence of current use of smoke tobacco in urban, or rural areas (Table 3).

#### Inadequate consumption of fruits and vegetables.

Overall, there was no change in the prevalence of inadequate consumption of fruits and vegetables, at 87.8% in 2014 and 86.4% in 2023 (p= 0.067). However, we observed a significant decrease in the prevalence of inadequate consumption of fruits and vegetables in the Eastern region from 91.7% in 2014, to 83.8% in 2023 (p< 0.001); whereas a significant increase was observed in the Eastern region from 75.1% in 2014 to 82.5% in 2023. No significant changes were observed in the prevalence of inadequate consumption of fruits and vegetables in urban, or in rural areas (Table 3).

#### Inadequate physical activity.

The prevalence of inadequate physical activity remained low at 5.0% in 2014 and 3.6% in 2023, overall there were significant changes between the two surveys (p= 0.003). Furthermore, we observed significant decrease between the two surveys in the prevalence of inadequate physical activity the Northern region from 3.9% in 2014 to 1.5% in 2023 (p= 0.003), and in the Central region from 7.5% in 2014 to 4.4% in 2023 (p= 0.003) (Table 2). A significant decrease in the prevalence of inadequate physical activity was also observed in rural areas from 4.2% in 2014 to 2.3% in 2023 (p=<0.001) (Table 3).

#### Sedentariness.

The prevalence of sedentariness significantly increased overall from 26.6% in 2014, to 31.9% in 2023 (p< 0.001). The significant increases occurred in all regions of the country except in the Eastern region (p= 0.226) – Table 2. Significant increases were also observed in rural areas, from 24.7% 2014, to 31.9% 2023 survey (p< 0.001) (Table 3).

#### High blood pressure.

Our analysis showed no overall significant change in the prevalence of high blood pressure between the 2014 survey at 24.6%, and that in the 2023 survey at 25.4% (p=0.418); neither were there significant changes between the two surveys in the four regions of the country (Table 2), nor by urbanicity (Table 3).

#### High blood glucose.

There was an overall significant increase in the prevalence of high blood glucose from 1.5% in 2014, to 3.3% in 2023 ((p< 0.001). Significant increases between the 2014 survey and the 2023 survey were also observed in all regions of the country, except in the Northern region (p=0.172)- Table 2. Significant increases in the prevalence of high blood glucose were also observed in rural areas from 1.3% in 2014 to 3.2% in 2023 (p< 0.001). No significant changes were observed in urban areas between the two surveys – Table 3.

#### Overweight and obesity.

There was an overall significant increase in the prevalence of overweight and obesity, from 19.3% in 2014 to 24.1% in 2023 (p< 0.001). The significant increase was observed in three regions of the country, except the Eastern region (p= 0.145). There was also a significant increase the prevalence of overweight and obesity in rural areas from 16.2% in 2014 to 19.9% in 2023 (p< 0.001). No significant changes were observed in urban areas.

## Discussion

Our analysis provides a comprehensive overview of the overall prevalence of the common NCD risk factors in Uganda. The results reveal two important findings; first, the prevalence of most of the common NCD risk factors increased between 2014 and 2023, including the prevalence of high blood pressure, high blood glucose, overweight and obesity, and current alcohol consumption. The most dramatic increase was the prevalence of high blood glucose which more than doubled from 1.5% in 2014 to 3.3% in 2023. Second, our findings show that the prevalence of some of the NCD risk factors remains unacceptably high including the prevalence of high blood pressure (> 25%), current alcohol consumption (> 20%), and inadequate consumption of fruits and vegetables (> 80%), even though the prevalence of some of these may not have changed significantly between 2014 and 2023. The high prevalence of these risk factors is equally important to pay attention to, aiming to reduce their prevalence.

The findings call for more actions to be taken aimed at not only curbing the increase, but also reducing the prevalence of the NCD risk factors to ultimately reduce the risk of high rates of NCD-associated morbidity, disabilities and mortality in the future. The World Health Organization has already warned about the rapidly increasing burden of NCDs within the African region [2]. It has also set global targets and indicators to monitor these [21,22], which provide reasonable guidance to countries. However, NCD risk-factor surveillance is yet considered a priority in many low- and middle-income countries [3].

We also note from our analysis that the overall prevalence of inadequate physical activity reduced significantly from 5.0% to 3.6%, and that of sedentariness reduced significantly from 26.6% in 2014 to 31.9% in 2023. Although the reduction in the prevalence of both may appear inconsistent, previous studies have shown that sufficient levels of moderate to vigorous physical activity and relatively high levels sedentary time can coexist in the same population. Owen et al (2010), Ford and Caspersen (2012) and Wilmot et al (2012) [23–25], have all reported that sufficient levels of moderate to vigorous physical activity do not preclude relatively high levels of sedentary time and vice versa. Thus, although the prevalence of inadequate physical activity in the Uganda population is currently relatively low and efforts should continue to keep it low, there is at the same time a need to take preventive measures aimed at curbing the increasing prevalence sedentariness.

We recognize the steps that  have been taken in Uganda aimed at curbing the increasing burden of NCDs in the country including: development of an NCD Multi-Sectoral Strategic Plan [10], launch of the presidential initiative on healthy eating and healthy lifestyles (Uganda Ministry of Health, 2017), and launch of a National Day of Physical activity - held annually every first Sunday of July to raise awareness about the growing number of NCDs in Uganda [11], and others like tobacco use control (tobacco taxes, advertising ban), increasing alcohol taxes and alcohol advertising restrictions, salt policies, and trans-fat policies. However, our findings indicate that these measures have not yet made a significant impact on the prevalence of most of the common NCD risk factors. It is possible that it is too early for these to have had a wider impact at population level, and/or there hasn’t been effective implementation of these. Whatever the case might be, there is a need to for the various stakeholders, led by Uganda’s Ministry of Health to periodically re-examine and evaluate strategies aimed at curbing the increasing burden of NCDs, including those that aim at reducing the prevalence of the common NCD risk factors. Indeed it has previously been noted that despite the availability of cost-effective and evidence-based practices to address NCDs in LMICs [26], implementing these interventions remains a significant challenge, particularly in promoting healthy behaviors [27].

Further, the 2030 Agenda for Sustainable Development Goals adopted by the United Nations in 2015 recognized NCDs as a major public health challenge. Specifically, sustainable Development Goal (SDG) number 3 includes target 3.4 to reduce premature NCD mortality by one-third by 2030 [28]. If this goal is to be achieved, concerted prevention actions are needed. NCDs continue to pose a serious public health concern in Uganda. For example in 2021 the age-standardized mortality rate across four major NCDs (Cardiovascular Disease, Chronic Respiratory Disease, Cancer and Diabetes) was 709 per 100,000 in males and 506 in females [29]. In 2019, NCDs contributed 36% of all deaths in the country. These statistics too, emphasize the need for the various stakeholders, including Uganda’s Ministry of Health, to identify and implement interventions aimed at reducing the prevalence of NCD risk factors to prevent the currently increasing burden of NCDs and associated morbidity and mortality that has already been noted by the WHO Africa Regional Office [2].

### Strengths and limitations

An important strength of our analysis is that it is based on data collected from nationally representative sample surveys. Thus, the prevalences reported represent a national picture. Second, the two surveys used the standardized WHO STEPS methodology, a methodology that has been validated and used in many LMIC countries, which makes the data reliable and the findings comparable.

We note two limitations to our study. First, data on the behavioral NCD risk factors was based on self-reported behaviors including current alcohol consumption, current tobacco use, physical activity, and sedentariness. Self-reporting of the behavioral characteristics is not an objective way to measure the variables of interest, and is likely to have some recall bias as respondents may not correctly remember some behavioral events. Self-reported behaviors are also likely to be influenced by intentional social desirability reporting that may not reflect the actual behaviors. Both recall bias and biased social desirability reporting are forms of information bias that may lead to either over or under-estimation, and it is always difficult to determine the direction of this type of bias, if any. Whichever the case might be, the WHO STEPwise approach to NCD risk factor surveillance has been validated and widely used in many LMIC countries, and has been found to generate reliable and to generate comparable results. We therefore feel that any recall bias in our study did not significantly affect the reliability of our findings.

Second, participants reporting to had been using other remedies for the treatment of either hypertension or diabetes, other than those prescribed by a health care worker, were considered not be on treatment for these conditions; because of lack of evidence regarding the efficacy of such remedies for treating hypertension or diabetes. It is possible that there could be some remedies that are able to control hypertension or diabetes to undetectable levels, in which case we could have under-estimated the prevalence of high blood pressure, and/or high blood glucose.

## Conclusions

We make the following conclusions from our analysis:

1] The overall prevalence of the following NCD risk factors increased significantly between 2014 and 2023: current alcohol use, high blood glucose, and overweight and obesity.
2] The overalll prevalence of the following NCD risk factors decreased significantly betwen 2014 and 2023: current use of smoke tobacco and inaadequate physical activity.
3] There were no statistically significant changes in the other NCD risk factors including current use of all forms of tobacco, inadequate consumption of fruits and vegetables, and high blood pressure.
4] Regardless of the change in prevalence between 2014 and 2023, the prevalence of the following NCD risk factors remain persistently high (above 20%): current alcohol use, inadequate consumption of fruits and vegetables, high blood pressure, and overweight and obesity.

## Supporting information

S1 Data Uganda 2014 NCD risk factor STEPS survey data. (DTA)

S2 Data Uganda 2023 NCD risk factor STEPS survey data. (DTA)
